# Effects of melatonin on rumen microorganisms and methane production in dairy cow: results from in vitro and in vivo studies

**DOI:** 10.1186/s40168-023-01620-z

**Published:** 2023-08-29

**Authors:** Yao Fu, Songyang Yao, Tiankun Wang, Yongqiang Lu, Huigang Han, Xuening Liu, Dongying Lv, Xiao Ma, Shengyu Guan, Yujun Yao, Yunjie Liu, Haiying Yu, Shengli Li, Ning Yang, Guoshi Liu

**Affiliations:** 1 College of Animal Science and Technology, China Agricultural University, Beijing, China; 2 Beijing Changping District Animal Disease Prevention and Control Center, Beijing, China; 3 Beijing General Station of Animal Husbandry, Beijing, China; 4 Zhejiang Chinese Medical University, Hangzhou, Zhejiang China; 5 Beijing Jingwa Agricultural Science and Technology Innovation Center, Beijing, China

**Keywords:** Melatonin, Methane, VFA, Rumen, Microorganism, Metabolism

## Abstract

**Background:**

Methane (CH_4_) is a major greenhouse gas, and ruminants are one of the sources of CH_4_ which is produced by the rumen microbiota. Modification of the rumen microbiota compositions will impact the CH_4_ production. In this study, the effects of melatonin on methane production in cows were investigated both in the in vitro and in vivo studies.

**Results:**

Melatonin treatment significantly reduced methane production in both studies. The cows treated with melatonin reduced methane emission from their respiration by approximately 50%. The potential mechanisms are multiple. First, melatonin lowers the volatile fatty acids (VFAs) production in rumen and reduces the raw material for CH_4_ synthesis. Second, melatonin not only reduces the abundance of *Methanobacterium* which are responsible for generating methane but also inhibits the populations of protozoa to break the symbiotic relationship between *Methanobacterium* and protozoa in rumen to further lowers the CH_4_ production. The reduced VFA production is not associated with food intake, and it seems also not to jeopardize the nutritional status of the cows. This was reflected by the increased milk lipid and protein contents in melatonin treated compared to the control cows. It is likely that the energy used to synthesize methane is saved to compensate the reduced VFA production.

**Conclusion:**

This study enlightens the potential mechanisms by which melatonin reduces rumen methane production in dairy cows. Considering the greenhouse effects of methane on global warming, these findings provide valuable information using different approaches to achieve low carbon dairy farming to reduce the methane emission.

Video Abstract

**Supplementary Information:**

The online version contains supplementary material available at 10.1186/s40168-023-01620-z.

## Background

Low carbon economic agriculture is the future goal of all countries over the world, especially for those countries having large numbers of cows and sheep. According to the Food and Agriculture Organization (FAO), the livestock sector accounts for 18% of global greenhouse gas emissions [1]. Particularly, the carbon emissions from beef and milk production account for 41% and 20% of the total emissions from animal husbandry, respectively [2]. Global warming potential (GWP) analysis shows that methane is 25 times more potent than carbon dioxide in terms of greenhouse warming effect per molecule, but its half-life in the atmosphere is much shorter (around 12 years) than that of carbon dioxide (CO_2_), which can remain in the atmosphere for 300 to 1000 years. Therefore, cutting methane emissions may achieve a relatively quick effect to reduce global warming [3, 4]. The rumen is a unique organ which distinguishes ruminants from other livestock, and it is the main site of methane production [5]. Methanogenic bacteria or archaea habited in rumen are the “machines” of methane production. These microorganisms either freely localize in the rumen fluid, attach to the surface of the protozoa or the hydrogenase bodies of the protozoa cytoplasm, or may be bound to other microorganisms and food particles as well as to the rumen wall [6–9]. The methane generated in rumen releases into air by burping. Therefore, methane emission from ruminants has considerable greenhouse gas effect on the environment.

On other hand, these anaerobic bacteria, archaea, ciliated protozoa, and fungi in rumen coexist and cooperate to break down cellulose, hemicellulose, carbohydrates, and others to provide energy and nutrients to the cows [10, 11]. Therefore, the abundance and stability of rumen microorganisms in dairy cows play a crucial role in their health and production performance [12, 13]. With the advancement of the knowledge on the microbiome, different approaches have been used to manipulate the rumen microbiota with the purpose of preserving their beneficial effects on animal health while reducing the greenhouse gas emissions [14]. Among them, antioxidant feeding seems to be a suitable approach since it can alter rumen microbes in dairy cows with limited side effects. For example, feeding cattle with tannic acid (TA), which has a similar antioxidant effect to vitamin E, reduced CH_4_ production, crude protein (CP) digestibility, and VFA [15]. In vitro study found that resveratrol addition reduced the abundance of *Methanobrevibacter* and thus reduced the methane production [16]. The spent coffee grounds (SCG) feeding also induces shifts in the ruminal bacterial community and alters the correlation networks among bacterial taxa and ruminal volatile fatty acid (VFA) probably due to the antioxidant properties of SCG as well as its high content of melatonin [17, 18]. In the cows with high dose of SCG feeding, the genera *Treponema*, *CF231, Butyrivibrio*, *BF331*, *Anaeroplasma*, *Blautia*, *Fibrobacter*, and *Clostridium* in their rumens were significantly enriched [19]. These findings suggested that antioxidant feeding could change rumen microflora and modify methane and VFA production in ruminants.

Melatonin (MT), an ancient antioxidant, was first discovered in 1958 by Lerner et al. from the bovine pineal gland [20]. Since then, MT is identified in almost all organisms, from bacteria, fungi, and plants to mammals [21]. MT is produced not only in the pineal gland but also in other organs and tissues including the gastrointestinal tract, brain, liver, kidneys, adrenal glands, heart, thymus, gonads, placenta, and uterus [22]. In addition to its anti-inflammatory, sleep-promoting, mood-improving, reproduction-regulating, and immune-enhancing activities, melatonin is a potent antioxidant [23]. Its antioxidant effects have been well documented [24]. Currently, several studies have revealed the potential effects of melatonin on bacterial infections. For example, melatonin supplementation in cows reduced elevated milk somatic cell counts induced by *Staphylococcus aureus or Escherichia* *coli* infections [25, 26]. The antibacterial effect of melatonin might relate to its ability to reduce intracellular substrates, lipid levels, and metal binding which are required for bacteria growth [27, 28]. Several studies have showed that melatonin could regulate the activity of gut microorganisms or their metabolites to improve physiological functions of gut and protect against various intestinal diseases [29–32]. These included the beneficial effects of melatonin on intestinal epithelial cell permeability, energy utilization, motility, bicarbonate secretion, and tight junctions to alleviate the irritable bowel syndrome, Crohn’s disease, and ulcerative colitis [29]. This is partially attributed to melatonin’s effects on intestinal morphological structure and distribution of enterotoxin-producing *Escherichia coli* in the intestine [29]. Melatonin not only altered the metabolism of the intestinal microbiota but also affected the composition of the intestinal microbiota [28, 30–32]. Therefore, more and more studies have focused on the regulatory effects of melatonin on the gut microflora which play the important role in health and diseases [33–36].

However, the effects of melatonin on rumen microorganisms either in the in vivo or in the in vitro conditions have been rarely reported. The rumen microorganisms are important for nutrition of ruminants and also are responsible for carbon emissions associated with greenhouse effect. Therefore, in this study, we have first established an in vitro rumen fermentation model to study the effects of melatonin on rumen microorganisms as well as their metabolism related to methane production. Then, results obtained from the in vitro rumen fermentation model were further validated in the cows. We attempt to uncover the potential mechanisms of melatonin on rumen microbiota metabolism and methane production via both the in vitro and in vivo rumen fermentation models. We expect that this initial study will provide new information for the application of melatonin in ruminants.

## Materials and methods

### Chemicals

Melatonin (Food Grade) (99.32%) used for animal study was purchased from Huanggang Hengxingyuan Chemical Co., Ltd. (Hubei province, China). The detail of melatonin used for animal study was shown in Table S1. The artificial saliva was prepared from 400 mL of distilled water, 200 mL of liquid A and 200 mL of liquid B, 0.1 mL of liquid C, 1 mL of resazurin solution (0.1%), and 40 mL of reduction buffer solution. After preparation, the artificial saliva was placed at 39 °C and filled with CO_2_ gas to adjust the pH value to 6.8. Liquid A was prepared from 5.7-g Na_2_PO_4_, 6.0-g KH_2_PO_4_, 0.6-g MgSO_4_·7H_2_O, and 1-L distilled water. Liquid B was prepared from 4.0-g NH_4_HCO_3_, 35-g NaHCO_3_, and 1-L distilled water. Liquid C was prepared from 13.2-g CaCl_2_·2H_2_O, 10.0-g MnCl_2_·4H_2_O, 1.0-g C_O_Cl_2_·6H_2_O, 8.0-g FeCl_2_·6H_2_O, and 100-mL distilled water. Reduction buffer solution was prepared from 625-mg Na_2_S·9H_2_O, 4-mL NaOH (1 mol/L), and 100-mL distilled water. Resazurin was purchased from Shanghai Aladdin Biochemical Technology Co. (Shanghai, China). The chemical reagents used for artificial saliva were purchased from China National Pharmaceutical Chemical Reagent Co. (Shanghai, China).

### Melatonin, CH_4_, VFA, and metabolites detection

Melatonin (≥ 99.8%, M5250), used as the standard for liquid chromatography tandem mass spectrometry (LC–MS/MS) detection, was purchased from Sigma Aldrich (St. Louis, MO, USA). Methanol (HPLC grade) was purchased from Thermo Fisher Scientific (Waltham, MA, USA). Acetonitrile (HPLC grade) was purchased from Thermo Fisher Scientific (Waltham, MA, USA). Hydrochloric acid was purchased from Shanghai Aladdin Biochemical Technology Co. (Shanghai, China). Formic acid (HPLC grade) was purchased from Shanghai Aladdin Biochemical Technology Co. (Shanghai, China). 2-Ethyl butyric acid was purchased from Shanghai Aladdin Biochemical Technology Co. (Shanghai, China). Ultrapure water with a resistance of 18.2 MΩ cm − 1 was purified using a Milli-Q system (Millipore, Bedford, USA).

### Animals

In the in vitro rumen fermentation model, the ruminal fluid of the cows was collected from ruminal fistulas from Sanshi cattle farm, Changping District, Beijing. Cows used in the in vivo experiments were from the Nankou Third cattle farm in Changping District, Beijing. The TMR (total mixed ratio) diet and nutrient composition for in vitro and in vivo experiments were shown in Table 1. All experiments were approved by the China Agricultural University Laboratory Animal Welfare and Animal Experimental Ethical Inspection Committee. The approved protocol number for the study was AW80802202-1–1.

**Table 1:** Ingredient composition and nutrient levels (DM basis) of diets

Ingredients	Percentage	Nutrient levels (%)	
Fine material	9.04	Phosphorus	0.7
Oats	8.02	Dry matter	46.5
Silage	57.01	Protein	18.4
Omelo^a^	1.06	Insoluble protein	12.78
Flaked corn	7.39	Rumen-bypass protein	5.12
Corn	6.55	NDF^b^	36.21
Alfalfa	2.53	Acid wash insoluble protein	1.55
Wet beer lees	8.40	ADF^c^	24.48
Total	100.00	Neutral wash insoluble protein	3.71
		Lignin	5.08
		Starch	21.89
		Fat	4.62
		Ash	8.08
		Calcium	1.05

^a^Omelo was a kind of protein

^b^ *NDF*, neutral detergent fiber

^c^ *ADF*, acid detergent fiber

Particle size of in vitro experiments, powdered

###  The construction of in vitro rumen fermentation model and experimental designs

Four Holstein cows with rumen fistulae served as rumen fluid donors. To control their food consumption, each cow was fed same amount of TMR (22 kg dry matter/day/cow). After milking and before feeding, the rumen fluids were collected at 9:00 am. The rumen fluid of four cows was pooled together and filtered through four layers of gauze and then placed in a thermos flask to be mixed thoroughly. The in vitro rumen fermentation model was performed using Heinz anaerobic fermentation bottles, each of which was a 150 mL consisting of 50 mL of artificial saliva and 0.5 g of cattle farm TMR feeding material as the fermentation substrate and mixed with 25 mL of well-mixed rumen fluid. The air in the fermentation flasks was replaced with nitrogen, and the Hennessey stopper was sealed immediately after the air was exhausted. A pre-vacuumed gas collection bag (Hedetech, China) (200 mL) was installed on top of the stopper. The mixture was fermented anaerobically at 39 °C in a constant temperature incubator to collect fermentation gas and fermentation liquid. In some Heinz anaerobic fermentation bottles, different concentrations of melatonin solutions were added at the beginning of fermentation. These included melatonin 10^−3^, 10^−5^, and 10^−7^ M groups, respectively. Fermentation times were set for 2 h, 4 h, 8 h, 24 h, and 48 h. Three times in vitro experiments with six technical replicates each were performed. Six replicates were set for each fermentation sample of different treatments. Based on the results from the dose-responsive study, the 10^−3^ M melatonin was selected for the following study. A 10^−3^ M melatonin was added to the fermentation vial every 24 h, and the fermentation continued for 72 h. In the daily melatonin solution addition to the fermentation bottle, a specific Hennessey rubber stopper plug was used to keep the anaerobic condition (this was illustrated in Fig. S1). Six replicates for each sample were used.

### The animal study and sample collection

Twenty Holstein cows without rumen fistulae were involved in this study, which were kept in semi-open activity yards together with individual neck clamp and feed trough. The general information for these cows was recorded, including body weight (683 ± 22.4 kg), lactation days (152 ± 12 day), milk yield of control group (41.9 ± 2.2 kg/day), milk yield of melatonin group (42.2 ± 2.3 kg/day), and parity (3.1 ± 0.8). To control their food consumption, each cow was given the same amount of TMR dry material (22 kg DM/d/cow), and the intake was also recorded daily. Based on the average volume of 67 L of the cow’s rumen [37] and the in vitro study’s melatonin concentration of 10^−3^ M which was selected for the most of the studies, the feeding dose of melatonin was calculated as 15 g/day for each cow (10^−3^ mol/L × rumen volume (67 L) × melatonin molar mass (232 g/moL) ≈ 15 g). The cows were divided into a melatonin-fed group (15 g/day) and a control group with 10 cows in each group, respectively. Cows in the experimental group were fed melatonin powder (same quality as the in vitro study) wrapped with digestible paper daily after milking at 16:00 pm, while cows in the control group were fed digestible paper only (we did not feed melatonin from the rumen sampling tube since melatonin was fed daily and rumen sampling tube was inserted only in the time of rumen fluid collection). The treatment last for 24 days. Pre-feeding for 3 days before the experiment and simulated collection of respiratory gas and rumen fluid was also performed to prime cows for subsequent experiments. Breathing gas, rumen fluid, milk and blood samples were collected after milking at 9:00 am on days 0, 7, 14, and 21, respectively. Milk was collected at morning milking. Rumen fluid, blood, and breathing gas were collected sequentially after morning milking and before morning feeding (breathing gas was collected from 5 cows in each group, as long constraint of cows would affect their performance).

Forty milliliters of milk was collected with preservative and stored at 4 °C for testing milk composition. Ten milliliters milk was collected without preservative stored at − 20 °C for melatonin determination. Ruminal fluid was collected from a rumen sampling tube with its metal end entering the cow’s mouth and into the rumen, and then the rumen fluid was sucked out using a 200-mL syringe (Fig. S2a). The first 20 mL of rumen fluid was discarded, and then 50 mL of rumen fluid was collected and stored at − 80 °C. Blood was collected from the tail vein. Five milliliters of blood was filled into EDTA anticoagulation tubes and stored at − 80 °C. Five milliliters of blood was placed in serum-separating tubes and centrifuged for the serum collection. The serum was stored at − 20 °C. Breathing gas was collected with gas respiration mask and gas bag (HedeTech, China) (50 L). The cattle respiration mask was purchased from China Agricultural University Beef Cattle Research Center (BRBC) (Fig. S2b). Simply, when the animal inhales, the outside gas enters the air inlet, and the outlet on the side of the exhaust port will be sealed, while when exhaling, the air inlet is sealed by a spring gasket, and the gas enters the ventilation pipe through the exhaust port into the breathing test bag. The breath test collection bag is made of heat-shrinkable polyurethane material for gas collection, with a size of 65 × 65 × 100 cm and a capacity of about 420 L. Before use, the gas bags were vacuumed. Fifteen seconds after the cows were put on the gas collection mask, the gas bag was connected to avoid residual gas in the gas collection mask tube to interfere test results. Breathing gas was collected for 10 min into a 420-L gas bag from each cow, mixed thoroughly, and then transferred to two 50-L gas bags to be stored for testing.

### Methane, VFAs, melatonin, milk composition, 16S rRNA sequencing, metagenomic sequencing, and metabolome detection in samples

#### Methane and VFA detection

Methane and VFAs in the samples were detected by Shimadzu 2010 gas chromatography (Japan). Five milliliters of rumen fluid was put into a plastic amperometric flask. Two-hundred milliliters of 1.0 mg/mL 2-ethylbutyric acid solution + 4 mL mixed solution of 1% hydrochloric acid and 5% formic acid were added. The mixture was shaken and mixed well. The mixture was placed in an ice water bath for 30 min and intermittently shaken and then centrifuged at 1500 rpm for 10 min. One milliliter of the supernatant was placed in a 1.5-mL centrifuge tube and centrifuged at 14,000 rpm for 10 min. The supernatant was taken again and passed through 0.45-μm filter and was ready for VFA detection. HP 19091N-213I (30 m × 0.32 mm × 0.50 μm) column was used for VFA detection. The injection volume for VFA detection was 1 μL. One mL of gas from the gas bag was drawn directly for methane detection. HP-PLOT/Q (30 m × 0.53 mm × 40.00 μm) column was used for methane detection. The carrier gas used for both the VFA and methane assays was *N*_2_ at a flow rate of 2 mL/min and 5 mL/min, respectively.

#### Melatonin detection

Melatonin in the samples was detected by Agilent 6470 liquid chromatography tandem mass spectrometry (USA). One milliliters sample (rumen fluid, serum, and milk) was mixed with 4-mL methanol. The mixture was vortexed for 10 min and placed at − 20 °C for 30 min. Finally, the sample was centrifuged for 10,000 rpm at 4 °C for 5 min. The supernatant was passed through a 0.22-μm syringe filter and was ready for MT detection. C18 reversed-phase column (50 mm × 2.1 mm × 1.8 μm) was used for melatonin detection. Gradient elution was performed using 0.1% formic acid water and methanol as the aqueous and organic phases, respectively. The flow rate was 0.4 mL/min. The injection volume was 2 μL.

#### Milk composition detection

Milk samples with preservatives were homogenized by ultrasound for 30 min at 37 °C for detection of milk composition. Milk compositions were detected by MilkoScan FT + (Denmark) automated tester for lactose, milk fat, milk protein, dry matter, and urea nitrogen in milk.

#### DNA extraction, PCR amplification, and 16S rRNA sequencing

DNA was extracted using E.Z.N.A. Stool DNA Kit (Omega Bio-tek, Inc., USA) following the manufacturer’s instruction. Concentration and quality of the genomic DNA were checked by NanoDrop 2000 spectrophotometer (Thermo Scientific Inc., USA). DNA samples were stored at − 20 °C for subsequent experiments. The V3–V4 region of the bacterial 16S rRNA genes was amplified using the primers 338F (5′-ACTCCTACGGGAGGCAGCAG-3′) and 806R (5′-GGACTACHVGGGTWTCTAAT′). For each sample, 8-digit barcode sequence was added to the 5′ end of the forward and reverse primers (provided by Allwegene Company, Beijing). Finally, universal primers with barcode sequences were synthesized and amplified on an ABI 9700 PCR system (Applied Biosystems, Inc., USA). The PCR was carried out on a Mastercycler Gradient (Eppendorf, Germany) using 25-μL reaction volumes, containing 12.5 μL 2 × Taq PCR MasterMix (Vazyme Biotech Co., Ltd., China), 3-μL BSA (2 ng/μL), 1-μL forward primer (5 μM), 1-μL reverse primer (5 μM), 2-μL template DNA, and 5.5-μL ddH_2_O. Cycling parameters were 95 °C for 5 min, followed by 28 cycles of 95 °C for 45 s, 55 °C for 50 s, and 72 °C for 45 s with a final extension at 72 °C for 10 min. The PCR products were purified using a Agencourt AMPure XP Kit (Beckman Coulter, Inc., USA). Sequencing libraries were generated using NEBNext Ultra II DNA Library Prep Kit (New England Biolabs, Inc., USA) following the manufacturer’s recommendations. The concentration and size of the library were assessed by NanoDrop 2000 (ThermoFisher Scientific, Inc., USA), Agilent 2100 Bioanalyzer (Agilent Technologies, Inc., USA), and ABI StepOnePlus Real-Time PCR System (Applied Biosystems, Inc., USA), respectively. The libraries were subjected to paired-end sequencing (2 × 300 bp) on Illumina MiSeq/NovaSeq 6000 (Illumina, Inc., USA) platform at Beijing Allwegene Technology Co., Ltd. The raw data was divided into different samples according to the barcode sequence through QIIME (v1.8.0) software [38]. Pear (v0.9.6) software [39] was used to filter and splice raw data. The sequences were removed from consideration if they were shorter than 120 bp, had a low quality of score (≤ 20), and contained ambiguous bases. During splicing, the minimum overlap setting was 10 bp, and the mismatch rate was 0.1. After splicing, sequences less than 230 bp in length were removed using VSEARCH [40] (v2.7.1) software, and chimeric sequences were removed by comparison with the Gold Database using the UCHIME [41] method. Qualified sequences were clustered into operational taxonomic units (OTUs) at a similarity threshold of 97% using UPARSE algorithm [42] of VSEARCH (v2.7.1) software. The BLAST tool [43] was used to classify OTU representative sequences of bacterial 16S sequences into different taxonomic groups against Silva138 [44] database, and e-value threshold was set to 1e-5.

#### Metagenomic sequencing analysis

The previously extracted DNA samples were fragmented to an average size of around 350 bp using Covaris M220 (Gene Company Limited, China) for paired-end (PE) library construction. PE library was constructed using TruSeq DNA Sample Prep Kit as per manufacturer’s instructions (Illumina). PE sequencing was performed using the Illumina HiSeq 4000 platform. Adapter sequences were removed from the 3′ and 5′ ends of the paired-end Illumina reads using SeqPrep (Version 1.1; https://github.com/jstjohn/SeqPrep). Low-quality reads (filtering of reads with sequencing adapters, filtering of reads with N (uncertain base) content ratio > 1%, filter reads with low quality bases (*Q* ≤ 20) content > 50%) were removed using Sickle (Version 1.33). Metagenomics data were assembled using default parameters of MEGAHIT (v1.0.6) [45]. Contigs with a length of over 500 bp were selected as the final assembly result. The contigs were then used for further gene prediction and annotation. Open reading frames (ORFs) from each assembled contig were predicted using prodigal software [46]. The predicted gene sequences were de-redundant at 0.95 similarity using CD-HIT software [47]. The nonredundant gene sets were obtained, and the sequencing data were compared with the constructed nonredundant gene sets using Bowtie software [48]. The information on the abundance of individual genes in different samples was counted and normalized to obtain the gene abundance table. Species composition analysis based on reads using diamond [49] compared to NCBI NR database combined with megan6 [50] parsing. The predicted nonredundant gene sets were compared with the functional annotation databases KEGG, COG/KOG, and GO [51–53], and the overall number of nonredundant genes annotated and the number in each sample were counted.

#### Metabolome analysis

Metabolome was measured by ultra-performance liquid chromatography tandem mass spectrometry (UPLC-MS/MS). Fifty-microliter sample was mixed with 300 µL of methanol. The mixture was vortexed for 3 min and centrifuged at 12,000 rpm for 10 min at 4 °C. Two-hundred microliters of supernatant was placed at − 20 °C for 30 min and then centrifuged at 12,000 rpm at 4 °C for 3 min. The 150 µL of supernatant was taken again and was ready for metabolome detection. Waters ACQUITY UPLC HSS T3 C18 column (100 mm × 2.1 mm × 1.8 µm) was used for metabolome detection. Gradient elution was performed using 0.1% formic acid water and 0.1% formic acid acetonitrile as the aqueous and organic phases, respectively. The flow rate was 0.4 mL/min. The injection volume was 2 μL. Analyst 1.6.3 software was used to process the mass spectrum data. MultiQuant software was used to open the sample off-machine mass spectrometry file. R software was used to analyze hierarchical cluster analysis (HCA) of accumulation patterns of metabolites among different samples. Fold change and VIP values of OPLS-DA model were combined to screen differential metabolites. The metabolites with fold change ≥ 2 and fold change ≤ 0.5 in the experimental group and the control group were considered to be significant difference.

### Statistical analysis

16S rRNA gene sequencing and metagenomics statistics data are presented as bar plots based on *P*-values derived from a Kruskal–Wallis test. Based on the OTU and its abundance results, the *α*-diversity index was calculated using QIIME (v1.8.0) software and plotted using R [54] (v3.6.0) software. The *β*-diversity distance matrix was calculated using QIIME (v1.8.0) software, and PCA analysis was plotted based on the distance matrix using R (v3.6.0) software. For taxonomic data, FDR correction of the *P*-values was conducted in using the default stats packages available in R (V3.6.0). The data were expressed as mean ± SEM. If the comparison is in the same group before and after treatment, paired *T*-test was used; otherwise, the one-way analysis of variance (ANOVA) was performed followed by multiple comparison or multiple *T*-test using IBM SPSS (21.0) software. *P* < 0.05 was considered significant difference.

## Results

###  The effects of melatonin on methane production and VFA in the in vitro rumen fermentation model

**Fig. 1:**
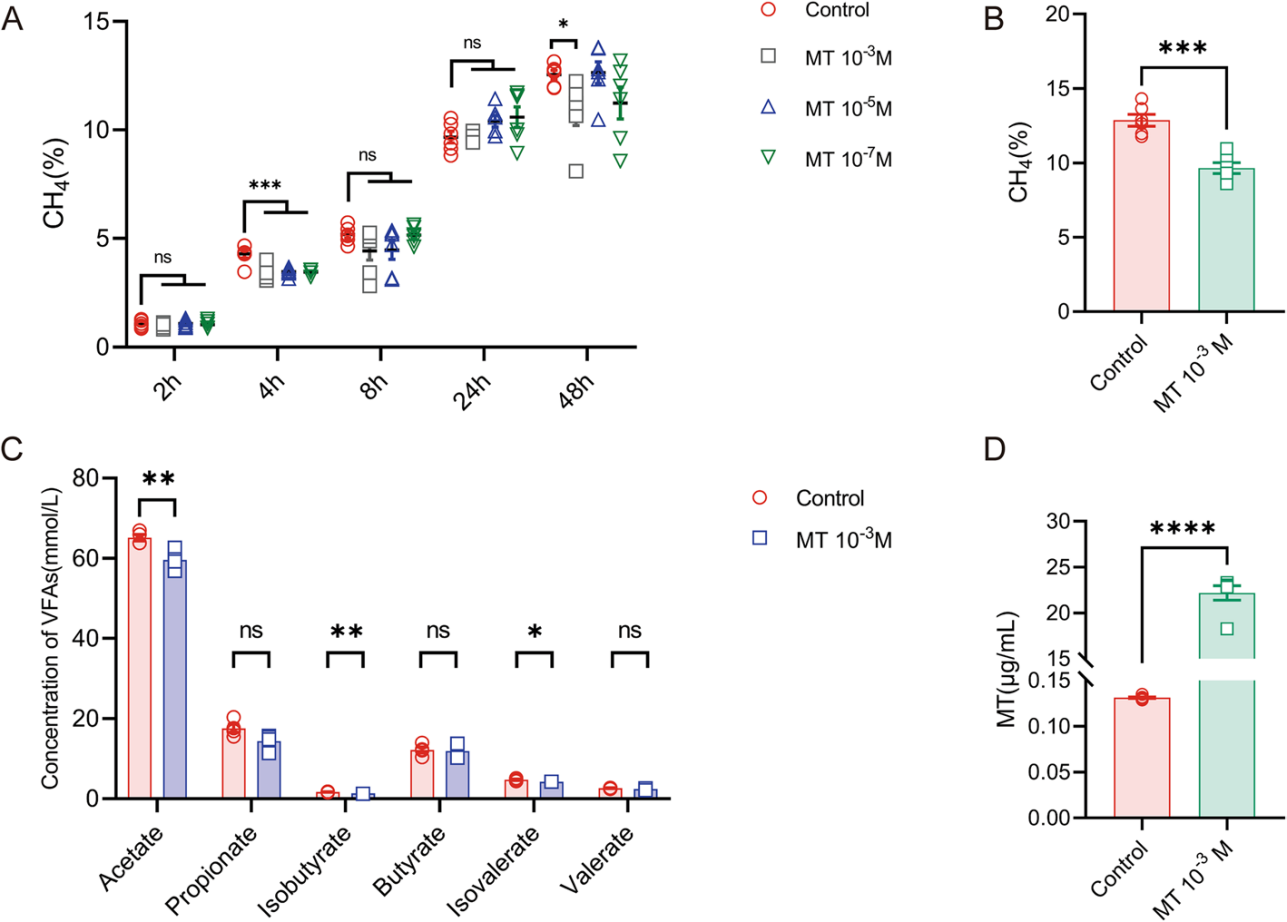
Effects of melatonin treatment on methane (CH_4_) production and VFA content in rumen in vitro fermentation model. **A** Methane (CH_4_) production at different concentrations and times. **B** Methane (CH_4_) production at 10^−3^ M concentration for 72 h of fermentation. **C** VFA content at 10^−3^ M concentration for 72 h of fermentation. **D** Melatonin levels at 10^−^.^3^ M concentration for 72 h of fermentation. Data were expressed as mean ± SEM (*N* = 6 for control, *N* = 6 for melatonin groups). * < 0.05, ** < 0.01, *** < 0.001

The methane content of all groups in culture flasks increased gradually from the period of 2 to 48 h incubations, indicating the success of the in vitro rumen fermentation model which exhibited active microbial fermentation to produce methane (Fig. 1A). The methane production was significantly lower at 4 h of fermentation in all melatonin groups than that in the control group (*P* < 0.001, Fig. 1A). Methane levels decreased by 20.3%, 19.18%, and 19.51% in the 10^−3^ M, 10^−5^ M, and 10^−7^ M melatonin groups, respectively, compared to the control group. Then, from 8 to 48 h period of fermentation, the methane content exhibited no significant differences among groups, excepting that 10^−3^ M melatonin group had a lower methane content than other groups (*P* = 0.032, Fig. 1A). This phenomenon suggested that melatonin might be exhausted by the metabolism of the rumen microbiota. Therefore, in the additional study, the melatonin was continuously (every 24 h) added into the fermentation system to keep a relatively stable melatonin concentration during the period of the study. The results showed that even at the 72-h fermentation, the melatonin level in MT-treated groups was significantly higher than that in the control group (*P* < 0.0001, Fig. 1D), and accordingly, the methane content in melatonin continuously treated groups was significantly lower than that in the control group (*P* < 0.001, Fig. 1B). The methane production in 10^−3^ M melatonin group was decreased 25.02% compared to the control group.

It had been reported that the abundance of rumen methanogens was positively correlated to the methane production [56]. These microorganisms degrade carbohydrates to produce VFA, which are the main source of energy for the ruminant organism [10]. The close relationship between methanogens and VFA was essential for maintaining normal rumen physiological function. To further confirm the effects of melatonin on rumen metabolism, the VFA content was examined. As shown in Fig. 1C, the melatonin treatment selectively decreased the concentration of some VFAs including acetate (*P* < 0.01, Fig. 1C), isobutyrate (*P* < 0.01, Fig. 1C), and isovalerate (*P* < 0.05, Fig. 1C) compared to the control group. Acetate is a short-chain fatty acid (SFA) that is the major substrate for de novo fatty acid synthesis [57]. A decrease in acetate is often accompanied by a decrease in milk fat.

###  Effects of melatonin on the composition of rumen microorganisms in vitro

**Fig. 2:**
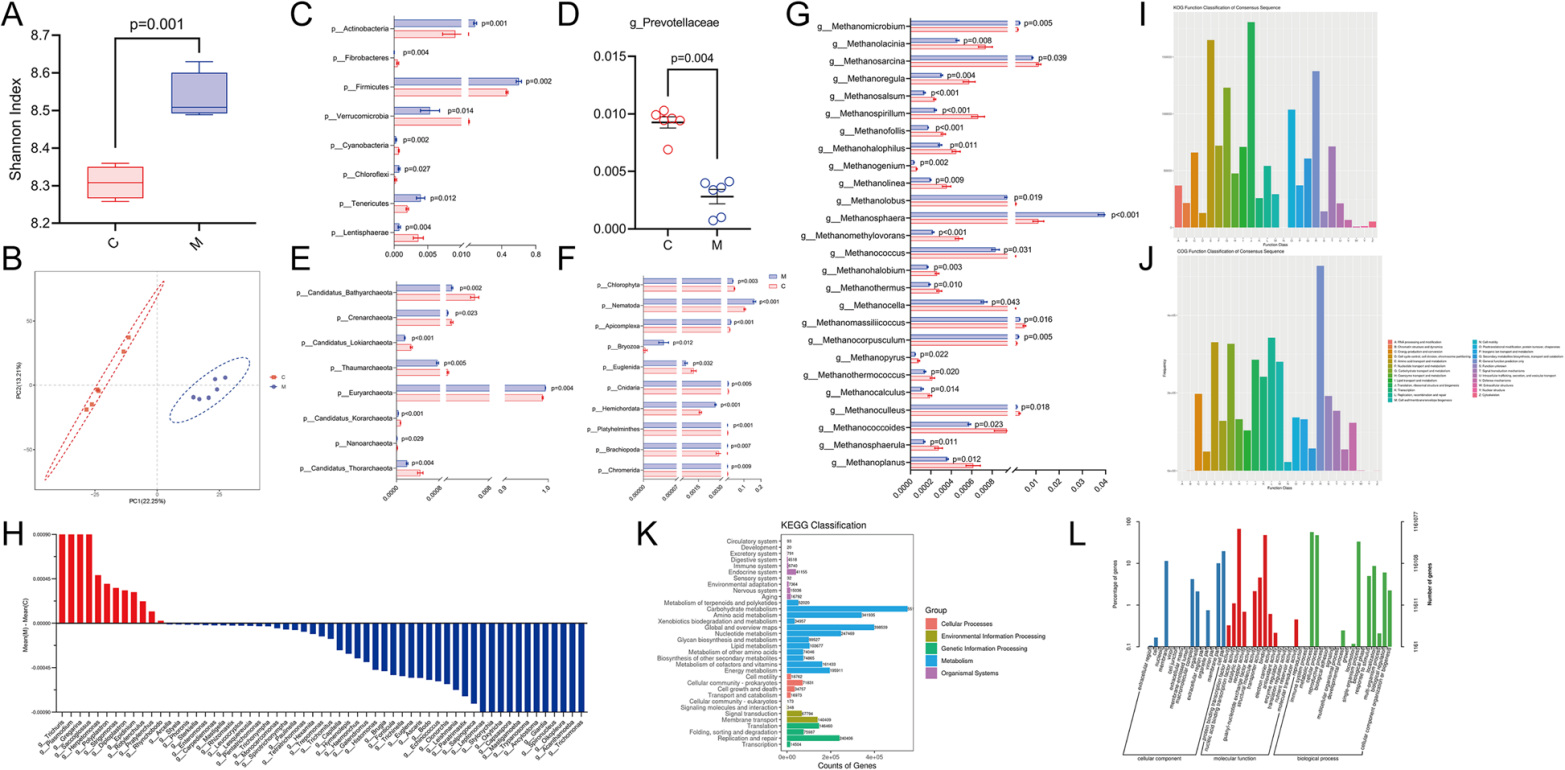
Effects of melatonin on rumen microbiota compositions and function feature change in the in vitro fermentation model. **A** Alpha diversity. **B** Beta diversity. **C** Differences in bacterial phylum levels by 16S rRNA sequencing. **D** Differences in the levels of *g_Prevotellaceae* by 16S rRNA sequencing. **E** Differences in *Archaea* phylum levels by metagenome sequencing. **F** Differences in fungi phylum levels by metagenome sequencing. **G** Differences in methanogens genus levels by metagenome sequencing. **H** Differences in protozoon genus levels by metagenome sequencing. **I** KOG (EuKaryotic Orthologous Groups) Function Classification of Consensus Sequence. **J** COG (Clusters of Orthologous Groups) Function Classification of Consensus Sequence. **K** KEGG (Kyoto Encyclopedia of Genes and Genomes) classification. **L** GO (Gene Ontology) classification. Data were expressed as mean ± SEM (*N* = 6 for control, *N* = 6 for melatonin groups). The *P*-values were identified on each of the graph

We carried out 16S rRNA gene sequencing to analyze the composition of rumen microorganisms. The results firstly showed that the alpha diversity in melatonin group was significantly higher than that of the control (*P* = 0.001, Fig. 2A), and a significant distinction in beta diversity between these two groups are also observed (Fig. 2B). The principal component 1 (PC1) and principal component 2 (PC2) explained 22.25% and 13.21% the variations, respectively. Due to the known VFA- and methane-reducing effect of melatonin, we focused potentially different influences of melatonin on rumen bacteria, archaea, or fungi. Analysis showed a significant differences in bacteria at the phylum level, between melatonin and control groups (Fig. 2C), in particular *Prevotellaceae* that are the major rumen bacteria to generate propionic acid [58]; we significantly reduced in abundance in melatonin group (*P* = 0.004, Fig. 2D), which was consistent with the simultaneously propionic acid reduction found in the study.

Archaea are the only known group of microorganisms that produce energy via methanogenesis [59], and archaea abundance is closely linked to methane production. We thus used metagenomic sequencing to analyze archaeal species composition of rumen microorganisms. The result showed that the melatonin-treated group significantly reduced the abundance of archaea phyla (*P* < 0.05, Fig. 2E). In detail, melatonin treatment led to significant changes in a total of 26 methanogenic genera; among them, 23 were significantly reduced, including g_*Methanoplanus*, g_*Methanococcoides* and g_*Methanospirillum*, etc. (all *P* < 0.05, Fig. 2G). Because of the symbiotic and interacting relationship between methanogenic bacteria, fungi, and protozoa in the rumen, the abundance of fungi and protozoa in rumen was also analyzed (Fig. 2F and H), and we found significant changes in total of 59 protozoa tested, among which 47 protozoa were significantly decreased (*P* < 0.05, Fig. 2H). Furthermore, rumen microbial metagenomic sequences were aligned to COG/KOG, KEGG, and GO databases to examine the effect of melatonin on rumen microbial function, and we found that all of them were mainly enriched in amino acid as well as carbohydrate transports and metabolisms (Fig. 2I and J). KEGG and GO analyses further demonstrate that rumen microbial differential genes were mainly enriched in carbohydrate metabolism, amino acid metabolism, catalytic activity, and metabolic process (Fig. 2K and L). In conclusion, melatonin has significant impact on rumen microbial function in dairy cows and likely affects their productive performance and metabolism.

### Effect of melatonin feeding on methane production and VFA in cows

**Fig. 3:**
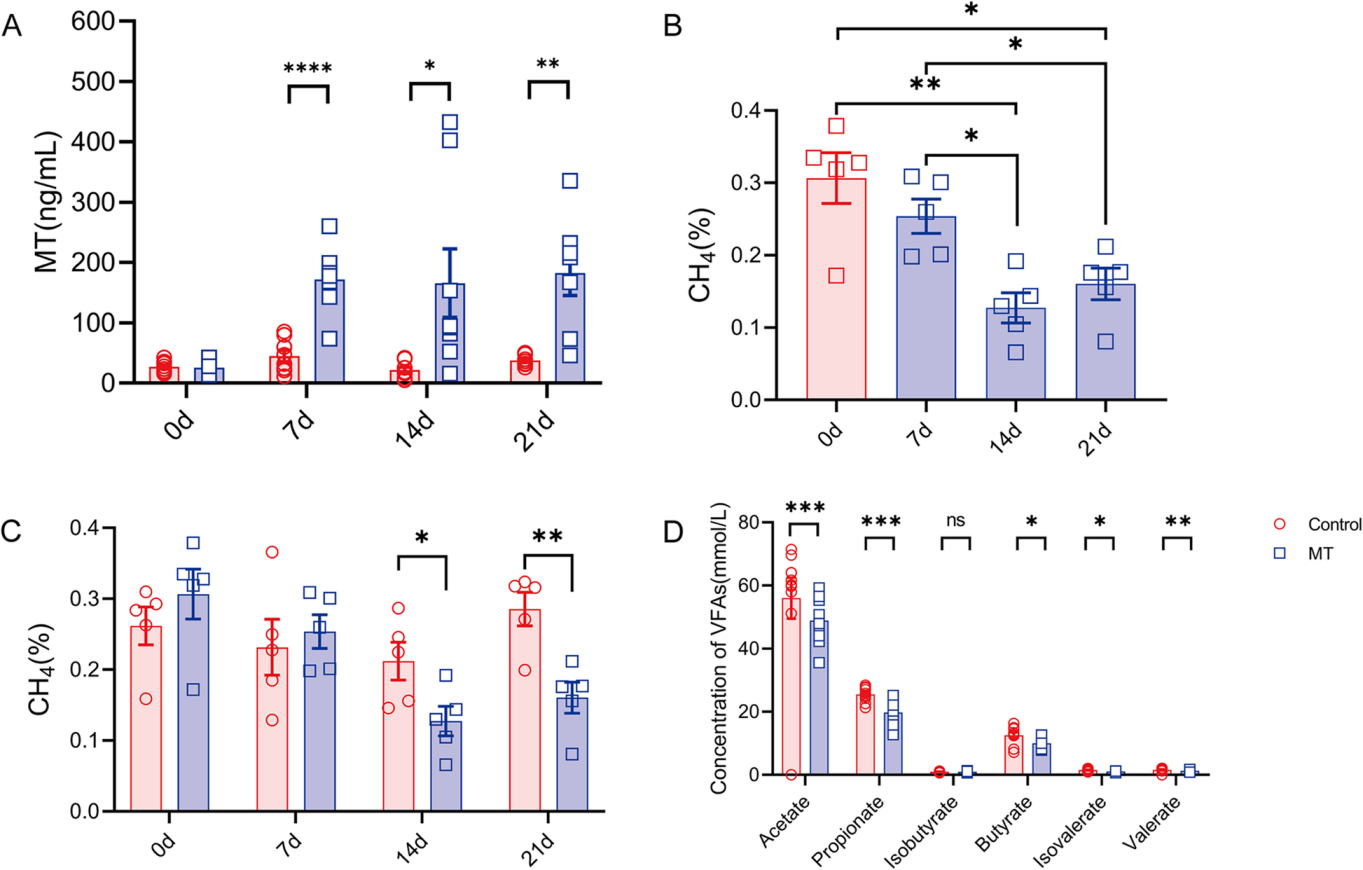
Effects of melatonin supplementation on rumen methane (CH_4_) production and VFA in cows with intact rumen. **A** Rumen fluid melatonin concentrations (*N* = 8 for control, *N* = 8 for melatonin groups). **B** CH_4_ production of melatonin-treated group compared with before and after treatment (*N* = 5 for control, *N* = 5 for melatonin groups). **C** CH_4_ production of melatonin treated compared with control group (*N* = 5 for control, *N* = 5 for melatonin groups). **D** VFA content. Data were expressed as mean ± SEM (*N* = 10 for control, *N* = 10 for melatonin groups). Ns, no significant difference, * < 0.05, ** < 0.01, *** < 0.001, **** < 0.0001

The result showed that melatonin was detected in the rumen fluid of all groups, indicating the naturally occurring melatonin is present in the rumen fluid. The results are consistent with Ouyang et al. [60]. They have reported the melatonin circadian rhythm in the rumen fluid of the cows. However, melatonin levels in the rumen fluid at 7, 14, and 21 days were significantly higher in melatonin group than that in control group (*P* < 0.01, Fig. 3A). Accordingly, the respiratory gases of the cows were also collected at 0, 7, 14, and 21 days after treatment. The results showed that methane content from respiration of the cows fed with melatonin significantly decreased from day 14 of the study compared to their baseline, i.e., decreased 53.03% at day 14 and 49.11% at day 21, respectively (*P* < 0.05, Fig. 3B). Meanwhile, the results also showed that there was a significant decrease of the methane emission from respiration of cows in melatonin group compared to the control cows at days 14 and 21, respectively (Fig. 3C). The VFA in ruminal fluid was measured at day 21 of feeding. The results showed that the VFA was significantly decreased in melatonin group compared to the control (*P* < 0.05, Fig. 3D). The significantly decreased VFAs included acetate, propionate, butyrate, valerate, and isovalerate. The trends of both methane and VFA changes in the in vivo experiment were consistent with the in vitro experiment.

### Effect of melatonin feeding on the composition of rumen microbiota of cows

**Fig. 4:**
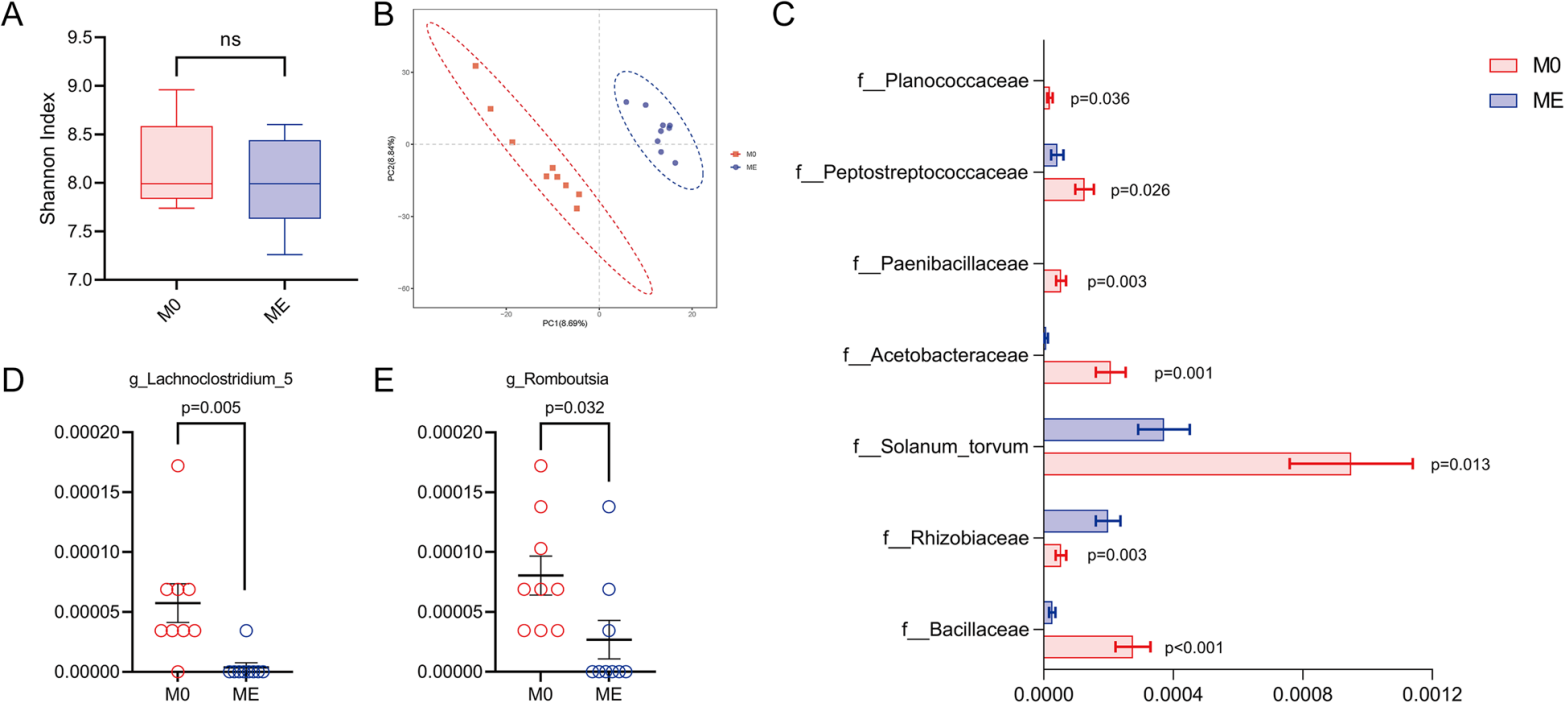
Effects of melatonin on distributions of microbiota in rumen of cows measured with 16S rRNA sequencing. **A** Alpha diversity. **B** Beta diversity. **C** The data of rumen microbiota at family level. **D** Differences in the levels of *g_Lachnoclostridium_5*. **E** Differences in the levels of *g_Romboutsia*. Data were expressed as mean ± SEM (*N* = 9 for control, *N* = 9 for melatonin groups). The *P*-values were identified on the each of the graph

16S rRNA gene sequencing was used to analyze the composition of rumen microorganisms between MT-feeding group and control. The results showed that the alpha diversity was not significantly different between melatonin and the control groups (Fig. 4A). This result was different from the in vitro study, and the exact mechanisms are currently unknown and requires further research. The results from the in vivo study indicated that melatonin did not alter the flora abundance in the intact rumen. As to the beta diversity, the PC1 and PC2 accounted for 8.69% and 8.84%, respectively, implying that the differences between the groups were significantly greater than that within the group (Fig. 4B). This difference presumably was due to the aggregated feature of the flora in melatonin group, indicating melatonin’s capacity to regulate the distribution of the microbiota in the rumen in the in vivo condition. The bacteria of rumen microflora that differed significantly at the family level were shown in Fig. 4C. The g_*Lachnoclostridium_5* and g_*Romboutsia* were significantly downregulated in melatonin group compared to the control group (Fig. 4D and E). *g_Lachnoclostridium_5* was involved in the metabolism of a variety of carbohydrates, and its fermentation produced acetate and butyrate [61]. G_*Romboutsia* was the main butyrate producer [62]. The decrease of these two bacteria was consistent with the decrease of rumen VFA in dairy cows.

### Effect of melatonin feeding on the metabolism of dairy cows

**Fig. 5:**
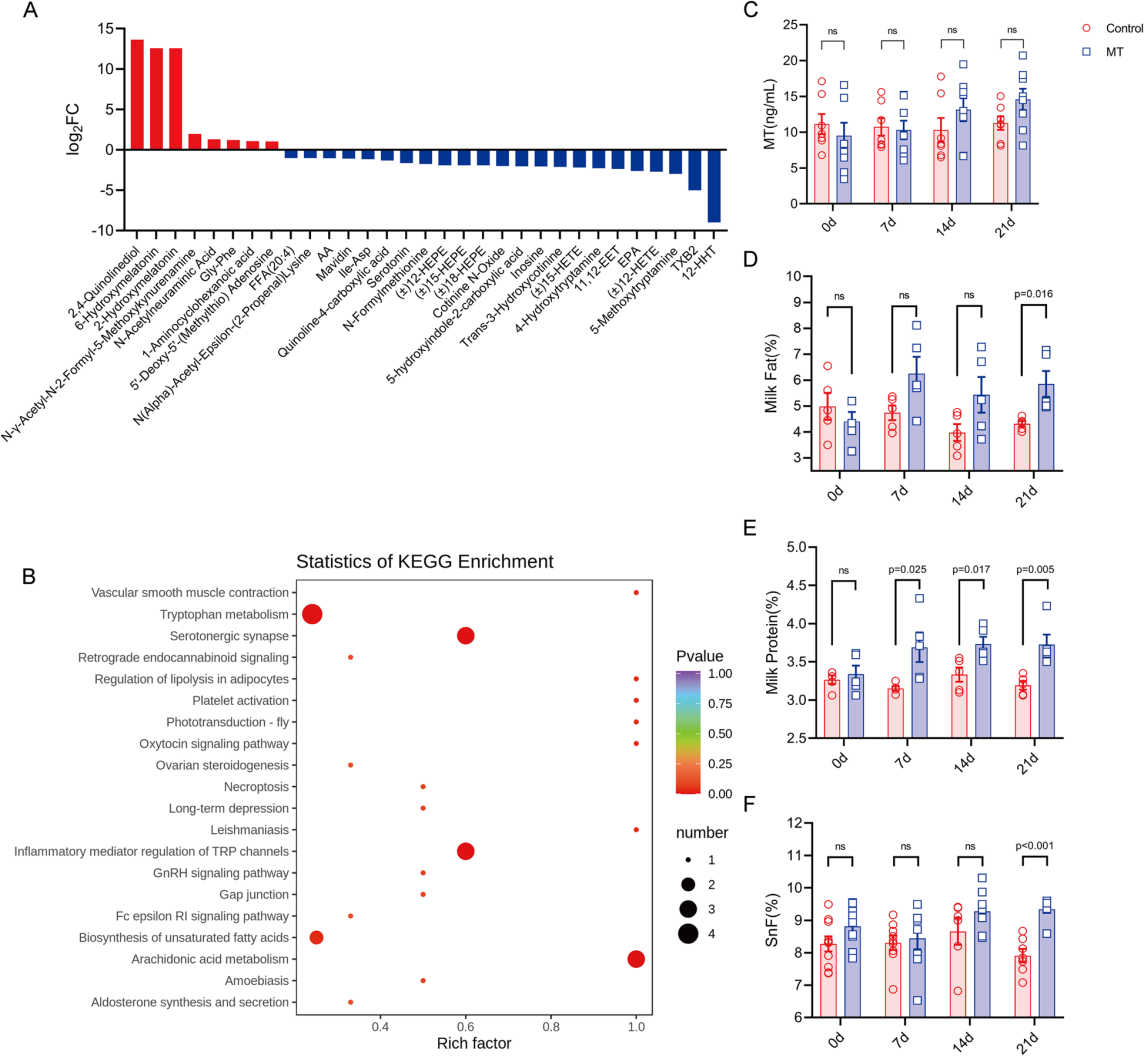
Effects of melatonin feeding on peripheral blood metabolomics and milk composition of dairy cows. **A** Differential metabolites. **B** KEGG enrichment analysis of differential metabolites. **C** Serum melatonin levels (*N* = 9 for control, *N* = 9 for melatonin groups). **D** Milk fat (*N* = 5 for control, *N* = 5 for melatonin groups). **E** Milk protein (*N* = 5 for control, *N* = 5 for melatonin groups). **F** Solids non-fat (*N* = 5 for control, *N* = 5 for melatonin groups). Data were expressed as mean ± SEM. The *P*-values were identified on the each of the graph

To ensure consuming the same amount of food, each cow was given TMR of 22 kg dry matter/day. The record showed that every cow completed its daily TMR, and no alteration of food intake was observed between the cows of melatonin treated and control groups. In addition, the body weight between the two groups is also stable. The results showed that melatonin reduced the production of VFAs seemly was not due to the food consumption but altered microbiota metabolisms. The regulatory effect of the melatonin-treated group on rumen microbial structure in the in vivo study was not as great as in the in vitro study. Likely, melatonin level in the rumen of the cow was not as high as in the in vitro study (10^−3^ M) as we calculated since the rumen melatonin could be distributed into the peripheral blood of the cow and metabolized. In order to verify this speculation, the peripheral blood of cows was collected for analysis of the melatonin level as well as the broad metabolomics. It was found that the serum melatonin level was slightly increased with time in melatonin group compared to the control group (Fig. 5C); however, the serum melatonin metabolites including 2-hydroxymelatonin and 6-hydroxymelatonin were significantly higher than those of the control, and this indicated that a portion of rumen melatonin was distributed to other tissues and degraded. In addition, a total of 31 differential metabolites were screened in serum by metabolome analysis, of which 8 were up- and 23 were downregulated in melatonin group compared to the control group (Fig. 5A). The amino acid metabolites including N-acetylneuraminic acid, N-γ-acetyl-N-2-formyl-5-methylkynurenine, and glycyl-phenylalanine (Gly-Phe) were significantly upregulated (Fig. S3e, c, and f) indicating the elevated amino acid metabolism, while metabolites associated with lipid oxidation including thromboxane B2 (TXB2), arachidonic acid (AA), and eicosapentaenoic acid (EPA) were significantly downregulated (Figs. S4d, c, and v). The differential metabolites were annotated and displayed using the KEGG database (Fig. 5B). The differential metabolites were enriched in metabolic pathways such as tryptophan metabolism, serotonergic synapse, inflammatory mediators, regulation of tryptophan channels, biosynthesis of unsaturated fatty acids, and arachidonic acid metabolism.

The milk composition of cows was also examined. The results showed that milk fat content was 16.28% higher at day 21 (*P* = 0.016, Fig. 5D); protein content was significantly higher at day 7 [17.07% (*P* = 0.025)], day 14 [12.00% (*P* = 0.017)]; and day 21 [16.73% (*P* = 0.005)], respectively (Fig. 5E); and the solids non-fat (SnF) was significantly higher (18.08%) at day 21(*P* < 0.001, Fig. 5F) in melatonin group than that in the control group. There were no significant differences in milk lactose and urea nitrogen between groups (Fig. S5A and B). Acetate is a major source of energy and substrate for milk fat synthesis in the dairy cow [63]. The results showed that acetate, butyrate (Fig. 3D), and metabolites associated with lipid oxidation (Fig. S4d, c, and v) decreased in the melatonin treated group, and their decline could result in the decrease of milk fat. Unexpected, the significant increase in milk fat on day 21 was observed, and this increase might be a result of the reduced methane production (see discussion). Milk protein increased significantly from day 7 compared with control group. Judging from the metabolome data, this might relate to the increased amino acid metabolism induced by melatonin (Fig. S3e, c, and f), thus improving the milk protein synthesis.

## Discussion

Ruminal microorganisms play an important role in the metabolism and health of the host, and the nutrients produced by rumen microorganisms meet up to 70% of the energy needs of the ruminants [10, 64]. Therefore, modulation of rumen microbial fermentation can improve the productive performance of the ruminants. In the rumen, the majority of the cellulose and hemicellulose are degraded to form VFAs for energy metabolism, while this process also produces some by-products including methane, water, and hydrogen [65]. Among the six short VFAs (acetate, propionate, isobutyrate, butyrate, isovalerate, valerate) generated in rumen, their compositions will impact the amounts of hydrogen production which serves as the substrate of methane synthesis. As a result, the fermentation pattern of rumen VFAs directly affects the methane production [65]. For example, supplementation with 3-nitrooxypropanol (NOP) significantly decreased total VFA content and ratio of acetate to propionate and reduced the methane production in ruminants [66]. Wang et al. [67] also reported that addition of polyphenols significantly reduced methane production due to the decreased acetate (A), increased propionate (P), and the decreased A/P ratio in the in vitro rumen fermentation model. Danielsson et al. also showed that alterations of the rumen fermentation products, such as the ratio of butyrate (B) and propionate (P) to total VFA, influenced methane production, indicating that methane formation depends on different fermentation pathways which are dependent on the compositions of the rumen microbiota. The formation of acetate and butyrate is associated with the production of methanogenic substrates, formate and H_2_. Usually, the high-yielding cows often have the high amounts of CH_4_ emission [68]. The current study has showed that melatonin treatment has reduced rumen methane production with the reduction of total and individual VFAs in the rumen. The decrease in VFAs can be caused by decreased food intake of the cows or also indicated a decrease in rumen metabolism of cellulose, H_2_ production by the ruminal microbiota. In the current study, the food consumption was carefully controlled, and no significant difference was observed between melatonin-treated and the control cows. Thus, the focus was given to the ruminal microbiota compositions and metabolism.

The compositions of ruminal microbiota directly influence methane production. The association between the methane production and rumen microbes has been extensively studied. Shabat et al. [69] reported that rumen microorganisms were variable in cows, and these variations caused different fermentation pathways with varied VFAs production, which in turn affected host’s fed-material utilization. Sheep with high methane emission exhibited the increased methanogenic pathways and upregulated gene expression related to these pathways in rumen microbiotas [70]. Wallace et al. [71] selected high and low methanogenic cattle to study their rumen flora composition in which the 16S/18S rRNA gene abundance analysis showed that the number of *Methanobrevibacter* in the rumen of high methanogenic cattle was 2.5-fold higher than that in the low methanogenic cattle. In addition, the methane production not only in rumen involved methanogenic bacteria but also in other non-methanogenic bacteria and some common bacteria, as well as protozoa; their genetic differences also affected methane production. For example, the abundance of *Vibrio succinogenes* family was fourfold lower in the high methanogenic group than the low methanogenic group. KEGG analysis showed that archaeal genes which were directly or indirectly responsible for methane production in cattle with high methane production were 2.7-fold more abundant than that in its low counterparts [71]. Succinivibrionaceae family was more abundant in cows with low CH_4_ production [68]. In the current study, we found that melatonin significantly reduced the abundance of most of the dominant rumen methanogenic bacteria and caused significant changes in a total of 26 methanogenic genera; among them, 23 were significantly reduced, including *g_Methanoplanus*, *g_Methanococcoides*, and *g_Methanospirillum bacteria*. In addition, melatonin caused significant changes in total of 57 of 59 protozoa tested; among them, the populations of 47 protozoa were significantly decreased. The dramatic reduction of methane production (in vitro around 25%, in vivo around 50%) by melatonin may be related to its inhibitory effect on the methanogenic genera and may also relate to that melatonin breaks the symbiotic relationship between these bacteria and protozoa. We also realized that the current data could not fully support that all the methane reduction effect was due to the melatonin’s effect on the alterations of the rumen microbiota by its antioxidant activity as mentioned in the introduction, and some other activities of melatonin might be involved, such as its bacterial static, metabolic regulatory, and immune activities [14]. But the relationships among melatonin administration, altered rumen microbiota, and methane reduction are indeed present. The exact mechanisms warrant for future studies. Beside impacting the compositions of rumen microorganisms, the potential effects of melatonin to modify the functions of rumen microbiota cannot be ignored since metagenomic results showed that rumen microbial differential genes were enriched in carbohydrates as well as amino acids transports and metabolisms in melatonin-treated group compared to the control. Therese processes are closely associated with energy metabolism of organisms. The exact role of functional alterations of rumen microbiota on the methane production after melatonin treatment cannot be distinguished from other factors at current study and requires well-designed future studies.

Interestingly, melatonin increased the abundance of beneficial bacteria including Ruminococcaceae, *Bifidobacterium*, and Lachnospiraceae (Fig. S6a, b, and c) while decreased the abundance of pathogenic bacteria such as *Romboutsia* (Fig. 4D), *Lachnoclostridium* (Fig. 4E), and *Citrobacter* (Fig. S6d). This observation suggests that melatonin selectively compromises some microbial activity but boosts others, particularly downregulating the activity of methanogenetic bacteria. Based on these observations, it appeared that melatonin could improve the rumen flora composition and most importantly to reduce methane production. The methane emission from ruminants has profound impact on the global greenhouse effect [72]. In the in vitro study, melatonin addition reduced methane production by around 25%, and in the in vivo study, melatonin administration reduced the methane emission from the cow’s respiration by approximately 50%. We also realized that this number (50%) might not be very accurate since the respiration gas collection only last for 10 min. The reason is that under the current respiration gas collection equipment (Fig. S2b), the cows can only be restricted for a short moment; otherwise, it will jeopardize their production performance. With the improvement of the equipment in the near future, the more accurate number will emerge. But the current measurement, at least, reflects the tendency that melatonin can significantly reduce the methane emission from ruminants’ respiration. All these in vitro and in vivo data strongly indicated that melatonin was a molecule which has the capacity to dramatically reduce the methane production from cows. If this observation has been confirmed by others or in other species of ruminants, the environmental impact of this findings warrants further investigation.

In addition, the ruminal flora differential genes were enriched in pathways of lipid metabolism after melatonin treatment. A few studies have found a correlation between intestinal methanogenic bacteria and lipid metabolism. In a study of human intestinal flora, *Methanobrevibacter* were found to be negatively associated with total fat content [73]. A correlation between methanogenic bacteria and intestinal lipid deposition was reported in intestinal flora of poultry [74]. Furthermore, the dairy metabolome analysis showed that melatonin treatment reduced most metabolites associated with lipid oxidation, which indicated more fat deposition in cows. This fat deposition might alter the level of fat in the milk. Unexpectedly, the significant increase in milk fat on day 21 was observed. This increase might be the result of methane reduction, i.e., the energy saved from methane synthesis was used for lipid production, but this is speculation and is deserved for future study to confirm.

The rumen microorganisms also affected milk protein content in cows. For example, rumen *Prevotella* by affecting amino acid metabolism increase the protein content of milk, while *Methanobrevibacter mellerae* were enriched in the rumen of low milk protein cows by utilizing the energy to generate CH_4_ [56]. These observations were consistent with our findings, i.e., the content of milk protein was significantly higher in melatonin-treated groups than that in the controls. The metagenomic results also supported this observation, i.e., the differential genes of rumen microorganisms were mainly enriched in amino acid transport and metabolism in melatonin-treated group compared to the control.

Most of studies to modify rumen flora compositions were through nutritional intervention. However, genetic factors of the host had a profound influence on the gut microbial composition. Evidence showed that different individuals responded differently when the diet was the same [75, 76]. The effects of the host’s genetic factors on the gut microbial compositions had been documented in mice [77–79]. In ruminants, correlation between host’s genetic factors and rumen microorganisms has also been observed. For example, the phenotypes of cattle including their food utilization rate and methane emission feature influence their rumen microorganism composition which exhibits potential heritability [80, 81]. Based on the observations that melatonin had the capacity to improve rumen microorganism composition and reduce ruminal methane production, it was possible genetically to establish the low carbon emission cow population by screening cows with high expression of melatonin synthetic genes and high rumen melatonin levels. This will be our future goal.

## Conclusion

Current study found that melatonin improved rumen microbiota composition. Particularly, melatonin significantly reduced rumen methanogenic microorganisms to lower the methane production which directly related to reduce the greenhouse effect. In addition, melatonin may also modify the functions of some microorganisms to reduce the VFA production which further strengthened its methane reduction activity. Reduction of VFA production will inevitably lower the efficiency of feeding utilization and impact the nutrition of the ruminants. In the current study, we have not detected significant nutritional issue including the body weight chance and milk composition. However, the increased milk quality with elevated milk protein content indicated an improved nutritional status of cows with melatonin treatment. It was speculated that the decreased VFA production might be compensated by the reduced methane production since the energy used for methane synthesis could be converted to synthesize milk fat and proteins. Our observations provide novel information which relates to low carbon dairy farming and improved production performance of cows.

### Supplementary Information

**Additional file 1: Fig. S1.** Schematic diagram of continuous fermentation in vitro. ** Fig. S2a.** Rumen sampling tube. **Fig. S3.** Up-regulated metabolites. **Fig. S4.** Down-regulated metabolites. **Fig. S5.** Milk lactose and urea nitrogen between groups. **Fig.S6.** Differences in rumen bacterial at genus level.

**Additional file 2: Table S1. **Analysis results of melatonin products.

## Data Availability

All raw data and sequencing information can be requested by contacting the corresponding author Guoshi Liu (gshliu@cau.edu.cn).
