# Correction: Effects of melatonin on rumen microorganisms and methane production in dairy cow: results from in vitro and in vivo studies

**DOI:** 10.1186/s40168-023-01706-8

**Published:** 2023-11-06

**Authors:** Yao Fu, Songyang Yao, Tiankun Wang, Yongqiang Lu, Huigang Han, Xuening Liu, Dongying Lv, Xiao Ma, Shengyu Guan, Yujun Yao, Yunjie Liu, Haiying Yu, Shengli Li, Ning Yang, Guoshi Liu

**Affiliations:** 1 College of Animal Science and Technology, China Agricultural University, Beijing, China; 2 Beijing Changping District Animal Disease Prevention and Control Center, Beijing, China; 3 Beijing General Station of Animal Husbandry, Beijing, China; 4 Zhejiang Chinese Medical University, Hangzhou, Zhejiang China; 5 Beijing Jingwa Agricultural Science and Technology Innovation Center, Beijing, China

**Correction: Microbiome 11, 196 (2023)**

**https://doi.org/10.1186/s40168-023-01620-z**

Following the publication of the original article [1], the author reported that Additional file 2 was incorrect. The correct additional file is inluded here and the original article has been updated.

### Supplementary Information

**Additional file 2:** **Table S1.** Analysis results of melatonin products.

## References

[1] Fu Y, Yao S, Wang T (2023). Effects of melatonin on rumen microorganisms and methane production in dairy cow: results from in vitro and in vivo studies. Microbiome.

