# Supplementary material for: Effects of melatonin on rumen microorganisms and methane production in dairy cow: results from in vitro and in vivo studies
Source: Microbiome. 2023 Aug 29;11:196. doi: 10.1186/s40168-023-01620-z (PMC10463863; doi:10.1186/s40168-023-01620-z)
Supplement: Supplementary file 3 — Additional file 2: Table S1. Analysis results of melatonin products. [file 40168_2023_1620_MOESM2_ESM.docx]

Table S1 Analysis results of melatonin products

| ITEM | STANDARD | RESULT |
| --- | --- | --- |
| Identification: Positive | Positive reaction | Positive reaction |
| Loss on drying | 1.0% MAX | 0.11% |
| Residue on ignition | 0.1% MAX | 0.09% |
| Chloride and Sulfate | 0.02%MAX | Conform |
| Individual impurity | 0.1% MAX | Conform |
| Total impurity | 1.0% MAX | Conform |
| Purity (HPLC) | 98.5%-101.5% | 99.32% |
| Melting point | 117-120℃ | 117.3-117.7℃ |
| Conclusion | COMPLY WITH THE REQUIREMENT USP42 | |
| Storage | Store in a well-closed container protected from light | |

USP42: United States Pharmacopoeia 42
